# Crystalloid volume versus catecholamines for management of hemorrhagic shock during esophagectomy: assessment of microcirculatory tissue oxygenation of the gastric conduit in a porcine model using hyperspectral imaging – an experimental study

**DOI:** 10.1097/JS9.0000000000001849

**Published:** 2024-07-08

**Authors:** Alexander Studier-Fischer, Berkin Özdemir, Maike Rees, Leonardo Ayala, Silvia Seidlitz, Jan Sellner, Karl-Friedrich Kowalewski, Caelan Max Haney, Jan Odenthal, Samuel Knödler, Maximilian Dietrich, Daniel Gruneberg, Thorsten Brenner, Karsten Schmidt, Felix C. F. Schmitt, Markus Alexander Weigand, Gabriel Alexander Salg, Anna Dupree, Henrik Nienhüser, Arianeb Mehrabi, Thilo Hackert, Beat Peter Müller, Lena Maier-Hein, Felix Nickel

**Affiliations:** a Department of General, Visceral, and Transplantation Surgery, Heidelberg University Hospital; b National Center for Tumor Diseases (NCT), NCT Heidelberg, a partnership between DKFZ and University Hospital Heidelberg; c German Cancer Research Center (DKFZ) Heidelberg, Division of Intelligent Systems and Robotics in Urology (ISRU); d Department of Anesthesiology, Heidelberg University Hospital; e German Cancer Research Center (DKFZ) Heidelberg, Division of Intelligent Medical Systems; f Faculty of Mathematics and Computer Science, Heidelberg University; g HIDSS4Health – Helmholtz Information and Data Science School for Health, Karlsruhe, Heidelberg; h Department of Urology and Urosurgery, University Medical Center Mannheim, Medical Faculty of the University of Heidelberg; i DKFZ Hector Cancer Institute at the University Medical Center Mannheim, Mannheim; j Department of General, Visceral and Thoracic Surgery, University Medical Center, Hamburg-Eppendorf, Hamburg; k Department of Anesthesiology and Intensive Care Medicine, University Hospital Essen, University Duisburg-Essen, Essen, Germany; l Department of Digestive Surgery, University Digestive Healthcare Center Basel, Switzerland

**Keywords:** esophagectomy, hemorrhage, hyperspectral imaging, porcine model, surgery, surgical data science, translational research

## Abstract

**Introduction:**

Oncologic esophagectomy is a two-cavity procedure with considerable morbidity and mortality. Complex anatomy and the proximity to major vessels constitute a risk for massive intraoperative hemorrhage. Currently, there is no conclusive consensus on the ideal anesthesiologic countermeasure in case of such immense blood loss. The objective of this work was to identify the most promising anesthesiologic management in case of intraoperative hemorrhage with regards to tissue perfusion of the gastric conduit during esophagectomy using hyperspectral imaging.

**Material and methods:**

An established live porcine model (*n*=32) for esophagectomy was used with gastric conduit formation and simulation of a linear stapled side-to-side esophagogastrostomy. After a standardized procedure of controlled blood loss of about 1 l per pig, the four experimental groups (*n*=8 each) differed in anesthesiologic intervention, that is, (I) permissive hypotension, (II) catecholamine therapy using noradrenaline, (III) crystalloid volume supplementation, and (IV) combined crystalloid volume supplementation with noradrenaline therapy. Hyperspectral imaging tissue oxygenation (StO_2_) of the gastric conduit was evaluated and correlated with systemic perfusion parameters. Measurements were conducted before (T0) and after (T1) laparotomy, after hemorrhage (T2), and 60 min (T3) and 120 min (T4) after anesthesiologic intervention.

**Results:**

StO_2_ values of the gastric conduit showed significantly different results between the four experimental groups, with 63.3% (±7.6%) after permissive hypotension (I), 45.9% (±6.4%) after catecholamine therapy (II), 70.5% (±6.1%) after crystalloid volume supplementation (III), and 69.0% (±3.7%) after combined therapy (IV). StO_2_ values correlated strongly with systemic lactate values (r=−0.67; CI −0.77 to −0.54), which is an established prognostic factor.

**Conclusion:**

Crystalloid volume supplementation (III) yields the highest StO_2_ values and lowest systemic lactate values and therefore appears to be the superior primary treatment strategy after hemorrhage during esophagectomy with regards to microcirculatory tissue oxygenation of the gastric conduit.

## Introduction

Highlights

- Hyperspectral-imaging (HSI) of the gastric conduit during esophagectomy could reliably identify malperfusion in case of hemorrhage in a porcine model.
- HSI could also identify volume-based circulatory support to be superior compared to catecholamine-only strategies regarding gastric conduit resuscitation and oxygenation.
- Spectral changes during hemorrhage and resuscitation were characteristic and highly similar between porcine models and patient data indicating transferability.
- HSI as microcirculatory monitoring could open up new opportunities for the guidance of haemodynamic management during hemorrhage and resuscitation and therefore increase patient safety.

To this day, operative radical resection of affected malign tissue, including lymphadenectomy, remains one of the core principles, especially in the treatment of esophageal cancer. Despite technological advancements and optimization of the operative approach, such as robotic preparation during the abdominal and robotic phases, oncological esophagectomy, therefore, remains a two-cavity procedure with high morbidity and mortality^1–5^. While numbers are heterogeneous, overall morbidity and major morbidity rates are reported as high as 39 and 20%, with anastomotic leakage rates at 8%, mortality rates at 2% as well as median ICU stay and length of hospital stay at 2 and 11.2 days, respectively^2^. Mean blood loss is described to be around 200–300 ml with varying degrees of significant differences across different surgical approaches^6^. However, complex anatomy and the proximity to major vessels such as the azygos vein continue to constitute a certain risk for massive intraoperative hemorrhage, with little to no data being reported on the incidence or impact of these hemorrhage events. Currently and consequently, there is no conclusive consensus on the ideal anesthesiologic countermeasure in case of such immense blood loss during esophagectomy.

With the risk of malperfusion and the resulting impairment of an organ already widely considered to be prone to hypoxic tissue damage^7^, extensive hemorrhage could have devastating effects on a newly formed gastric conduit that has just experienced manipulation during preparation, resulting in suboptimal blood supply conditions. Gastric conduit malperfusion regardless of its local or systemic origin is one of the major driving factors behind anastomotic insufficiency^8^, a major complication in esophageal surgery yielding a significant increase in mortality rates for this procedure^9,10^. Consequently, a quick and decisive intraoperative reaction through the anesthesiological team beyond surgical measures is essential for the clinical course and fate of the patient.

While there are several options in the treatment of hemorrhagic shock including all kinds of circulatory supplement agents as debatable as colloidal fluids, primarily four approaches should be taken into consideration according to current medical knowledge. These four approaches are (I) permissive hypotension (i.e. temporarily tolerating insufficient blood pressure levels), (II) catecholamine therapy using noradrenaline, (III) crystalloid volume supplementation, and (IV) combined crystalloid volume supplementation with noradrenaline therapy. Although these options are established approaches to hemodynamic therapy in the event of circulatory compromise, success of recovery is often only measured based on macrocirculatory surrogate parameters such as mean arterial pressure (MAP)^11,12^, while the actually relevant capillary perfusion effects on a microcirculatory level are neglected due to a lack of objective evaluation methods in clinical routine. Laser speckle contrast imaging, laser Doppler imaging or ICG imaging are experimental approaches with promising concepts, but are far away from clinical routine^13^ and data on tissue effects of aforementioned therapy options (I–IV) on a microcirculatory level is scarce.

Hyperspectral-Imaging (HSI) as a new optical imaging technique has proven to be a promising tool regarding tissue oxygenation assessment already^14,15^. Using the differences in reflectance spectra caused by varying reflectance properties of various tissues, conclusions concerning organ state and characteristics can be made. Through computation of color-coded-images and in-depth spectrum analysis based on this data, gastric tissue oxygenation can be visualized and objectively quantified^15,16^. This nourishes the deliberation that HSI might be well-suited to assess and compare the effects of different anesthesiologic interventions. The aim of this study was, therefore, to evaluate the effects of different anesthesiologic interventions on the oxygenation of the gastric conduit during hemorrhage using HSI. The optimization of oxygenation assessment and intraoperative countermeasures following hemorrhage will help to better understand and control the clinical consequences of hemodynamic changes caused by the most common interventions currently used in hemorrhage treatment.

## Material and methods

### Porcine model, anesthesia, and monitoring

The experiments were approved by the Committee on Animal Experimentation of the regional council Baden-Württemberg in Karlsruhe (G-261/19). All pigs were managed according to German laws for animal use and care and according to the directives of the European Community Council (2010/63/EU) and ARRIVE guidelines^17^ (Supplemental Digital Content 1, http://links.lww.com/JS9/D34). Results from a smaller subgroup with a different scientific question have been published by Dietrich *et al*.^18^. Regular pigs (Sus scrofa domesticus) were used as an experimental nonsurvival animal model as per highly standardized and established institutional standard regarding handling and narcosis^16,18–23^. Pigs were deprived of food 24 h prior to surgery with free access to water. Initial sedation was performed with intramuscular injection of azaperone (Stresnil 40 mg/ml by Elanco) with 6 mg/kg (≈6 ml=240 mg) 15 min prior to further manipulation. Next, analgosedation was established by intramuscular injection of midazolam (Midazolam-Hameln 5 mg/ml by Hameln pharma plus gmbh) with 0.75 mg/kg (≈6 ml=30 mg) and ketamine (Ketamin 10% by Heinrich Fromme) with 10 mg/kg (≈4 ml=400 mg). Animals were ventilated in volume-controlled mode (Respirator: Primus Dräger Medical AG) with a tidal volume of 8 ml/kg, a positive-end-expiratory pressure of 5 mbar, an inspiration-to-expiration ratio (I:E ratio) of 1:2, and a fixed FiO_2_ of 0.5. The respiratory rate was adjusted to reach an end-tidal CO_2_ of 40±5 mmHg. Intraoperative anesthesia was achieved through balanced narcosis with sevoflurane and the combination of i.v. 0.2 mg/kg/h midazolam (≈1.5 ml/h=7.5 mg/h) and 8.75 mg/kg/h ketamine (≈3.5 ml/h=350 mg/h) at a rate of 5 ml/h. No relaxant agents were applied. Two peripheral venous catheters in the ear veins for pharmaceutical application, a three-lumen central venous catheter in the jugular vein for controlled hemorrhage plus volume therapy and an arterial line in the femoral artery for invasive blood measurement were established. Body temperature was sustained with electrical heat blankets and monitored with an esophageal temperature probe. An extensive effort was taken to standardize and control as many relevant external factors as possible and monitor the ones that could not be controlled. Confounding factors that were controlled include age, weight, housing conditions, body temperature, narcotics, ventilation, blood loss, and time management. Confounding factors that were closely monitored in order to guarantee homogeneity between the individuals include metabolism using blood gas analysis, circulation using Pulse Contour Cardiac Output Monitoring (PiCCO) and therapy surveillance by board-certified anesthesiologists. Monitoring included capnometry, electrocardiography, pulse oximetry using a saturation probe fixed to the tail, and invasive blood pressure measurement.

### Surgical procedure and experimental groups

**Figure 1:**
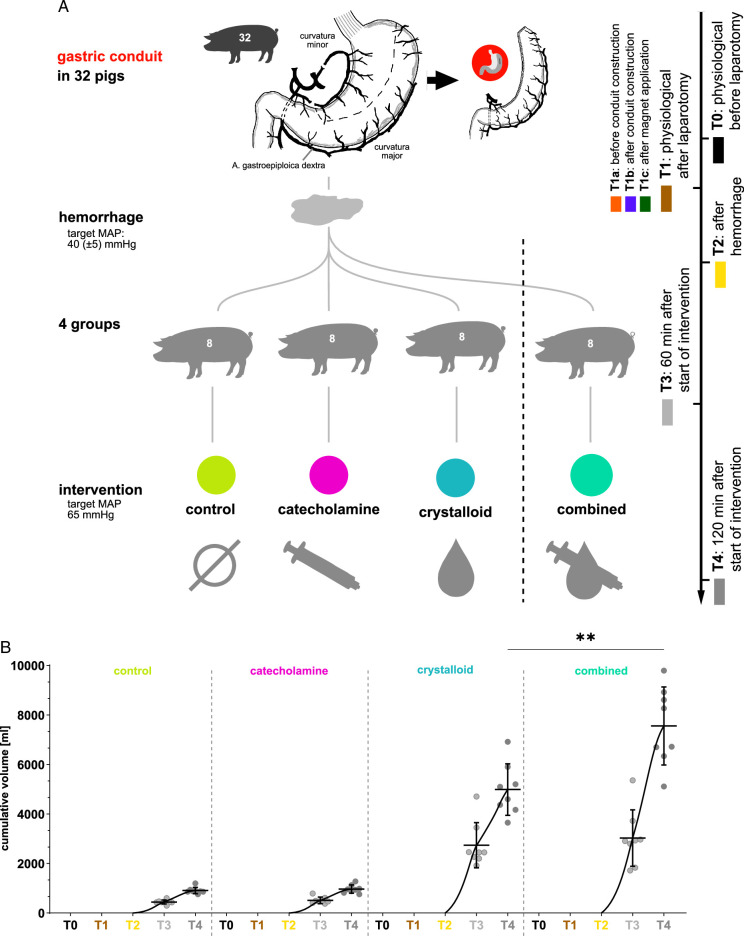
Recording protocol. A, visualization of the recording protocol with four groups along five timepoints. B, quantification of applied intravenous fluid.

A total of 32 animals were used for the experiments with a mean weight of 35.1 kg. Animals were randomly assigned into four different anesthesiologic intervention groups with eight animals each, that is, (I) permissive hypotension, (II) catecholamine therapy using noradrenaline, (III) crystalloid volume supplementation, and (IV) combined crystalloid volume supplementation with noradrenaline therapy with eight animals each (Fig. 1). Groups I–III therefore represent principal treatment strategies investigating singular and extreme effects, while group IV represents the most realistic treatment strategy in a clinical real-world scenario. All data from all animals was included in the analysis.

Following midline laparotomy, the stomach was mobilized and exposed and a gastric conduit was constructed using linear staplers. The only remaining vessels of supply were the right gastroepiploic artery and vein. There are different possibilities to perform the esophagogastric anastomosis. In general, it can either be performed by hand or stapler. In the case of the stapler, there are techniques using a circular stapler, mainly derived from rectal anastomotic techniques, and using a linear stapler, mainly derived from bariatric and upper gastrointestinal surgery. In the case of this study, the distal part of the linear stapler side-to-side anastomosis during minimally-invasive Ivor–Lewis esophagectomy was simulated with a linear magnet with the same dimensions (6 cm×0.5 cm) as a linear stapler in order to allow gastric conduit evaluation with HSI.

The procedure was therefore performed in an open fashion via midline laparotomy, but with the technical aspects as used in minimally-invasive esophagectomy.

After completing the surgical procedure, hemorrhagic shock was induced by drawing blood from the central venous catheter. The amount of blood removed was adjusted to achieve a target MAP of 40±5 mmHg for 60 min and aggregated to about 500 ml per animal. This amount is obviously large, but definitely realistic for intraoperative cumulative hemorrhage during esophagectomy, for example, in case of iatrogenic laceration of the thoracic caval vein or aorta. All animals received a basic fluid supplementation of 10 ml/kg/h crystalloid infusion (Sterofundin ISO by B. Braun). The control group (I) did not receive any further specific hemodynamic treatment regimen. The catecholamine group (II) received a continuous perfusor infusion of noradrenaline (Arterenol, Sanofi-Aventis Deutschland GmbH). The crystalloid volume supplementation group (III) received a continuous crystalloid infusion (Sterofundin, B. Braun SE). In both II and III, a MAP of 65 mmHg was set as a therapy target for the treatment period. Crystalloid fluid volume and catecholamine dose were titrated and recorded by the attending anesthesiologic experimenter to reach the aforementioned MAP targets. Experiments for group I–III with their fundamental and singular extreme effects were conducted initially in order to test feasibility when designing group IV^18^. As it became apparent that with this setup only a MAP of 60 mmHg could be reached reproducibly, the target MAP for group IV was also set at 60 mmHg in order to guarantee direct comparability between the principal treatment strategies of group I–III with the combined treatment strategy in group IV as the focus of this work was to see which treatment (with or without noradrenalin) would be beneficial for the conduit perfusion given equal MAP levels. Group IV then received crystalloid volume supplementation combined with noradrenaline therapy at a fixed perfusor rate of 1 μg/kg/min for noradrenaline (about 10 ml/h for a 35 kg pig using a 1:5 dilution of 1 mg/ml noradrenaline as perfusor solution) and a dynamically adjusted crystalloid infusion rate until a MAP of 60 mmHg was reached. Upon reaching this target pressure, additional crystalloid volume was only supplied when noradrenaline infusion alone proved to be insufficient in maintaining a MAP of 60 mmHg. Applied volume was precisely documented in Figure 1b and Supplement Table 1 (Supplemental Digital Content 2, http://links.lww.com/JS9/D35). Timepoints for HSI recordings and circulation parameter measurements, including mean arterial blood pressure, heart rate, hemoglobin, and systemic lactate, were:

1. T0: before laparotomy.
2. T1: after laparotomy.
  - T1a: before gastric conduit construction;
  - T1b: after gastric conduit construction;
  - T1c: after magnet application for linear stapler simulation.
3. T2: 60 min after hemorrhage.
4. T3: 60 min after intervention (group-specific).
5. T4: 120 min after intervention (group-specific).

After surgery, pigs were euthanized with a rapid i.v. application of 50 ml of potassium chloride solution. Death was pronounced upon an end-expiratory CO_2_ partial pressure below 8 mmHg.

To the best of our knowledge, this hemorrhagic gastric tube model was valid towards the actual condition in the patient and there were no intraoperative reasons to question the validity of the model and the similarity to situations that could be encountered during surgery on real patients.

Data from this porcine study was compared to a human dataset of 57 recordings across 10 individuals for physiological human gastric conduit and 14 recordings across 4 individuals for malperfused human gastric conduit recorded during the SPACE trial^24^.

### Hyperspectral imaging

**Figure 2:**
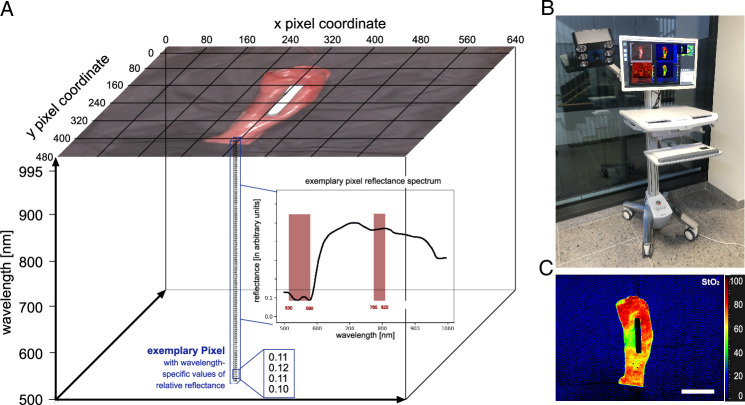
Hyperspectral Imaging technology. Visualization of HSI technology. A, HSI datacube with indicated spectral bands relevant for computing StO_2_. B, TIVITA Tissue camera system. C, example StO_2_ index image. Scale bar equals 5 cm.

HSI data was acquired with the TIVITA Tissue Halogen system from Diaspective Vision GmbH (Fig. 2). It provides a spectral resolution of 5 nm in the range from 500 nm to 995 nm. Six integrated halogen lamps provide a standardized illumination. Numerical values stored in the datacube represent reflectance values at every single pixel for every wavelength in arbitrary units. The computed tissue parameter index images include the hyperspectral oxygenation index (StO_2_) and underlying formulas can be reviewed in cited literature^25^.

HSI measurements were conducted on the gastric conduit. The region of interest in each HSI image was manually annotated using a polygon tool. Mean and SD reflectance spectra were obtained by calculating the median spectrum over every pixel within the annotated region of interest of one image, the mean spectrum over every image within one pig and then the mean over all pigs per group. Spectra are presented as indicated with either original or L1-normalized values on pixel level.

### Informatics and statistical analysis

All code was developed and executed with PyCharm 2019.1.2 and Python 3.7. Data visualization and basic statistical analyses were performed with GraphPad Prism 8.3.1. A *P*-value <0.05 was conventionally considered statistically significant, but all significance levels should be perceived descriptively. Statistical testing was done with multiple comparison testing using ordinary one-way ANOVA for unpaired parametrically-distributed data and mixed-effects analysis with Geisser-Greenhouse correction for paired parametrically-distributed data with assumed equal SDs. Kruskal–Wallis test was used for unpaired nonparametrically-distributed data, while Friedman test was used for paired nonparametrically-distributed data. Numerical values are provided with SD in brackets. Significance levels were adjusted for multiple testing and were indicated with * for *P*≤0.05, ** for *P*≤0.01, *** for *P*≤0.001, **** for *P*≤0.0001, and n.s. for ‘not significant’. Scale bars always equal 5 cm if not stated otherwise. Graphs depict mean and SD. A principal component analysis (PCA)^26^ was used to visualize the spectra in reduced dimensions. The trend of change in the spectra over time was visualized with a PCA computed on all L1-normalized spectra from T2 (hemorrhage induced) and T4 (120 min after intervention) and subsequently showing the trend between time points by an arrow in the reduced dimensionality space. Owing to the technical setup, all relevant confounding factors – as listed above – could be controlled and were homogenous during monitoring so that no adjustment in the statistical regression analysis was required or expedient and would have only worsened comprehension of the general readership. Consequently, univariate regression (also known as simple linear regression) was chosen as the form of regression analysis due to its simpler and more intuitive interpretation, focusing on the relationship between the single predictor and the outcome as would be relevant in the clinical scenario. We have tried to reduce bias as much as possible, by showing extensive data including nonaccumulated data whereever possible. No datapoints were filtered out and we provided the numerical values for the boxplot quantifications. All analyses were done with internal validation through several assessors.

## Results

### Baseline stomach and gastric conduit data (T1 and T2)

**Figure 3:**
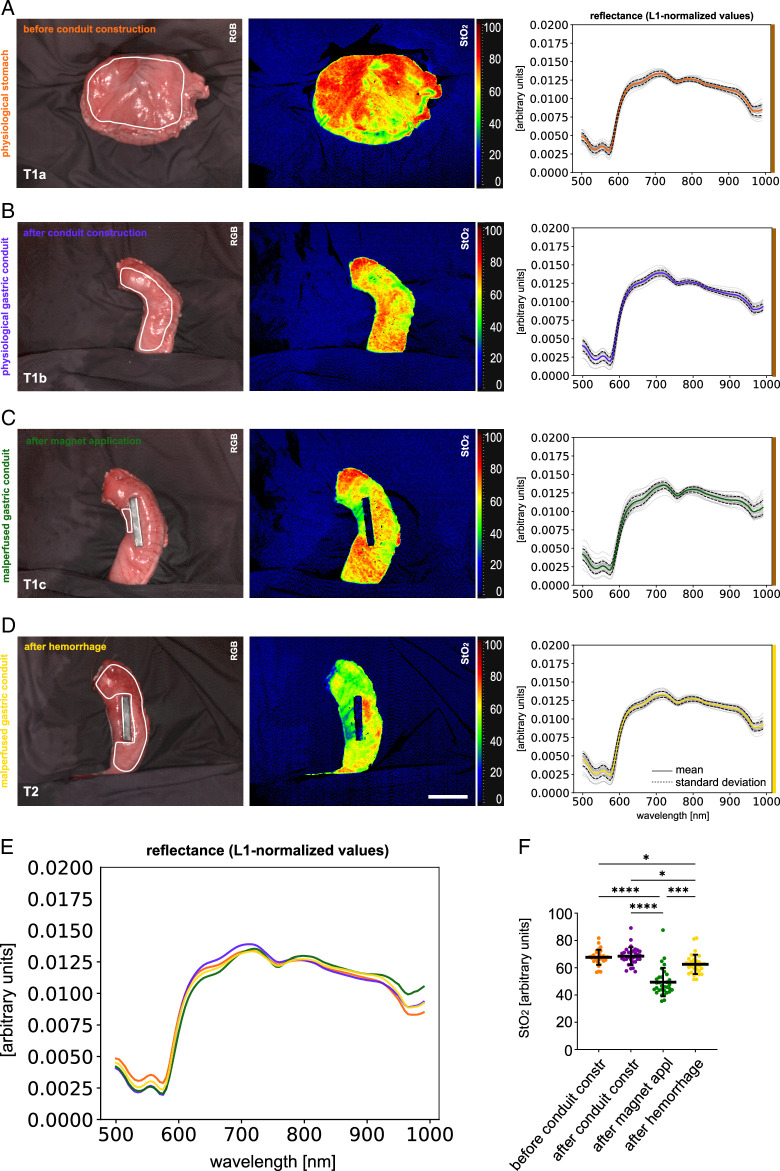
Visualization of physiological baseline stomach and gastric conduit data. HSI color index pictures and respective spectra in a porcine model (*n*=32). A, T1a: before gastric conduit construction. B, T1b: after gastric conduit construction. C, T1c: malperfused region of gastric conduit after magnet application (separate region). D, T2: gastric conduit after hemorrhage. E, comparison of L1-normalized reflectance spectra. F, quantification of hyperspectral index values for StO_2_. Scale bar equals 5 cm.

Different states of stomach and gastric conduit had different characteristic reflectance spectra (Fig. 3 and Supplement Figure 1, Supplemental Digital Content 3, http://links.lww.com/JS9/D36). StO_2_ values were different with 67.6% (±5.5%) for T1a (physiological stomach before gastric conduit construction), 68.5% (±6.5%) for T1b (after gastric conduit construction), 49.4% (±10.4%) for T1c (a separate malperfused region of the gastric conduit after magnet application), and 62.5% (±7.2%) for T2 (gastric conduit after hemorrhage) (Supplement Table 2, Supplemental Digital Content 4, http://links.lww.com/JS9/D37). It should be noted that the malperfused region of the gastric conduit after magnet application is a separate region that was only measured in order to evaluate the nadir the StO_2_ oxygenation values could reach in case of not only hemorrhage-induced hypoperfusion, but complete absence of perfusion.

### Differences in oxygenation of the gastric conduit

**Figure 4:**
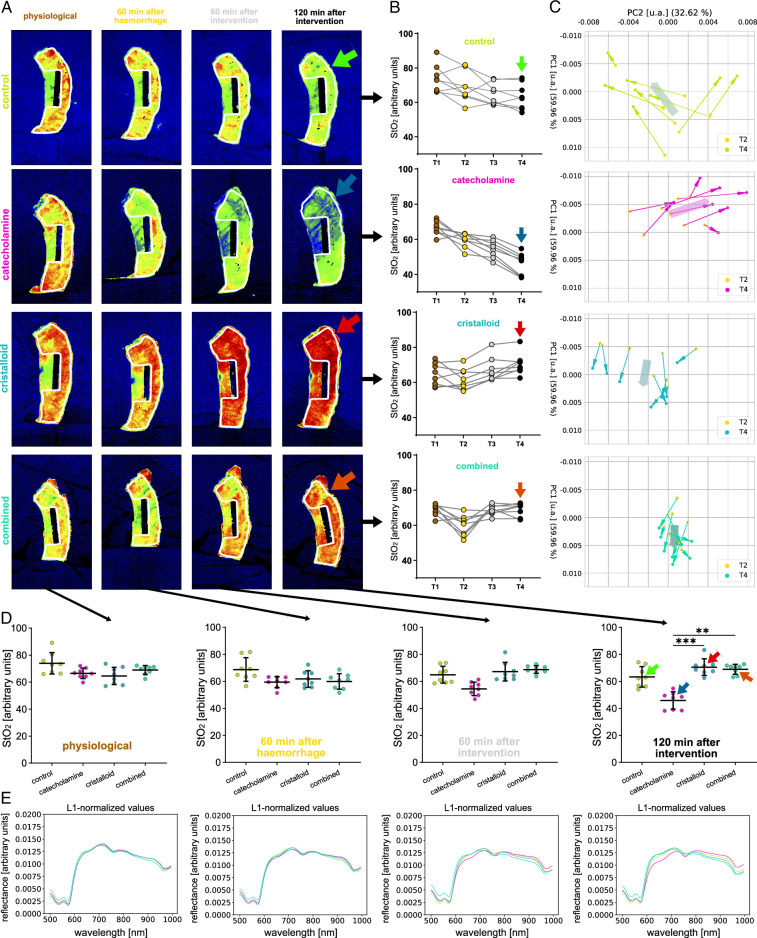
Temporal course of changes in reflectance of the gastric conduit (T1, T2, T3, T4). HSI color index pictures, StO_2_ quantifications and respective spectra for the gastric conduit over the course of baseline (T1), after hemorrhage (T2), 60 min after intervention (T3) and 120 min after intervention (T4) stratified for interventional groups. A, HSI StO_2_ color index pictures. B, StO_2_ quantifications and respective spectra comparing groups. C, PCA visualizing the development from T2 to T4. D–E, StO_2_ quantifications and respective L1-normalized reflectance spectra comparing timepoints stratified for interventional groups. Scale bar equals 5.

StO_2_ values and reflectance spectra of the gastric conduit were highly similar across all four experimental groups regarding baseline (T1) and hemorrhage (T2) measurements. However, StO_2_ values and reflectance spectra greatly differed across groups after 120 min of intervention (T4) with 63.3% (±7.6%) in the control group (I), 45.9% (±6.4%) in the catecholamine group (II), 70.5% (±6.1%) in the crystalloid group (III) and 69.0% (±3.7%) in the combined therapy group (IV) (Fig. 4, Supplement Table 3, Supplemental Digital Content 5, http://links.lww.com/JS9/D38, Supplement Figure 2, Supplemental Digital Content 6, http://links.lww.com/JS9/D39 and 3, Supplemental Digital Content 7, http://links.lww.com/JS9/D40). A PCA (Fig. 4C) showed distinctive trends for the four experimental groups at T4, especially showing that the directions of the trend of principal components of the cristalloid and combined group are similar, and different to the other two groups.

### Monitoring of circulatory physiology parameters

**Figure 5:**
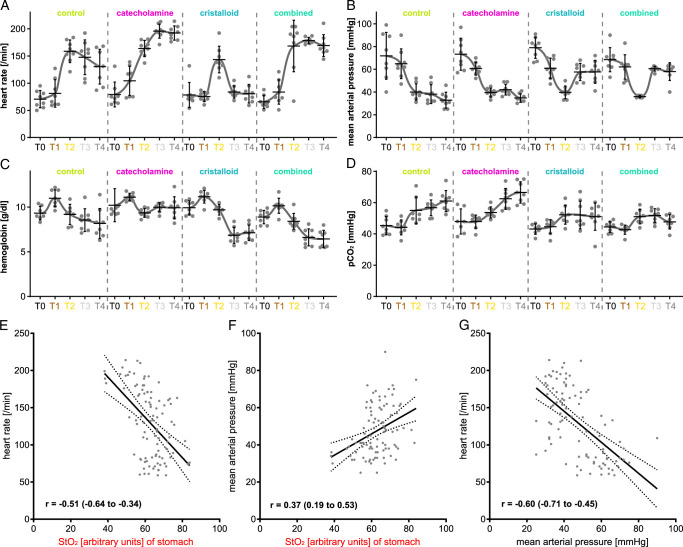
Circulatory physiology parameter values. A, heart rate. B, mean arterial blood pressure. C, hemoglobin concentration. D, pCO_2_. E, regression model for StO_2_ values of the stomach and heart rate (R^2^: 0.2565; y-intercept: 299.2 (SE: 30.06); slope: −2.707 (SE: 0.4753); F: 32.44; df: 1, 94; *P*: <0.0001). F, regression model for StO_2_ values of the stomach and mean arterial blood pressure (R^2^: 0.1401; y-intercept: 11.52 (SE: 9.261); slope: 0.5730 (SE: 0.1465); F: 15.31; df: 1, 94; *P*: 0.0002). G, regression model for mean arterial blood pressure and heart rate (R^2^: 0.3571; y-intercept: 0.228.6 (SE: 14.22); slope: −2.086 (SE: 0.2887); F: 52.22; df: 1, 94; *P*: <0.0001).

Heart rate, MAP, hemoglobin concentration, and venous pCO_2_ were monitored throughout the experiments and showed clear trends (Fig. 5 and Supplement Table 4, Supplemental Digital Content 8, http://links.lww.com/JS9/D41). With the knowledge that the combination of reduced heart rate, increased blood pressure and decreased pCO_2_ constitute the physiological state, it became apparent that group II (catecholamine) is comparably inferior regarding organ physiology as group I (control with permissive hypotension) with heart rates as high as 192.3/min (±13.1/min), MAP as low as 34.8 mmHg (±3.7 mmHg) and pCO_2_ as high as 66.5 mmHg (±6.8 mmHg). In comparison to this group III (cristalloid volume supplementation) yielded physiological parameters with heart rates at 80.5/min (±17.0/min), MAP at 57.8 mmHg (±10.1 mmHg) and pCO_2_ at 51.1 mm Hg (±8.8 mmHg). Interestingly, group IV (combined therapy) did reach the target MAP of 60 mmHg, but still presented clearly inferior physiology values compared to group III with heart rates at 169.1/min (±20.0/min), MAP at 58.0 mmHg (±7.8 mmHg) and pCO_2_ at 47.7 mmHg (±4.6 mmHg). StO_2_ correlated with heart rate and MAP to some extent.

### Validation of StO_2_ values using systemic lactate

**Figure 6:**
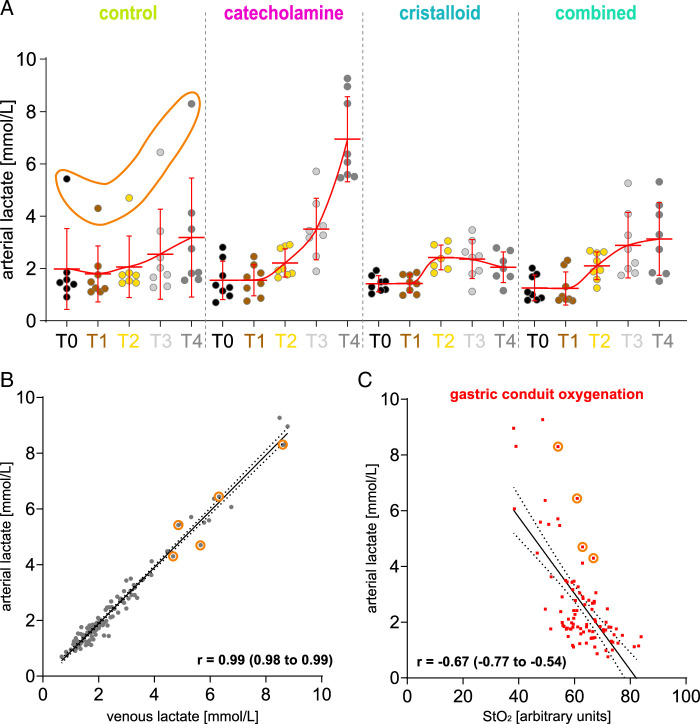
Corresponding lactate values. Depiction of lactate values of the experimental groups. A, lactate values (*n*=156). B, regression model of arterial and venous lactate values (R^2^: 0.97; y-intercept: −0.1238 (SE: 0.04747); slope: 1.007 (SE: 0.01537); F: 4290; df: 1, 114; *P*: <0.0001). C, a regression model for gastric conduit and arterial lactate (R^2^: 0.4459; y-intercept: 11.25 (SE: 1.010); slope: −0.1370 (SE: 0.01601); F: 73.22; df: 1, 91; *P*: <0.0001. Orange markings depict the outliers of an individual with impaired circulation. Scale bar equals 5 cm.

All groups initially reported highly similar levels of arterial lactate with 1.5 mmol/L at baseline (T1) and an increase to 2.1 mmol/L during hemorrhage (T2) averaged across groups. While both the control (I) and combined (IV) groups showed a moderate increase of lactate to 3.2 mmol/L (±2.3 mmol/L) and 3.1 mmol/L (±1.4 mmol/L) respectively, lactate in the catecholamine group (II) rose to 6.9 mmol/L (±1.6 mmol/L) 120 min after Intervention (T4), whereas lactate in the crystalloid group (III) declined to 2.0 mmol/L (±0.6 mmol/L) (Fig. 6, Supplement Table 5, Supplemental Digital Content 9, http://links.lww.com/JS9/D42). StO_2_ showed a distinctive negative correlation with arterial lactate with r=−0.67 (Fig. 6C). There was one outlier pig with constantly increased arterial lactate values. Interestingly, this individual also featured constantly decreased StO_2_ values, hereby actually positively contributing to the degree of correlation and validating StO_2_ values as prognostic markers per se.

### Comparison of porcine and human HSI data

**Figure 7:**
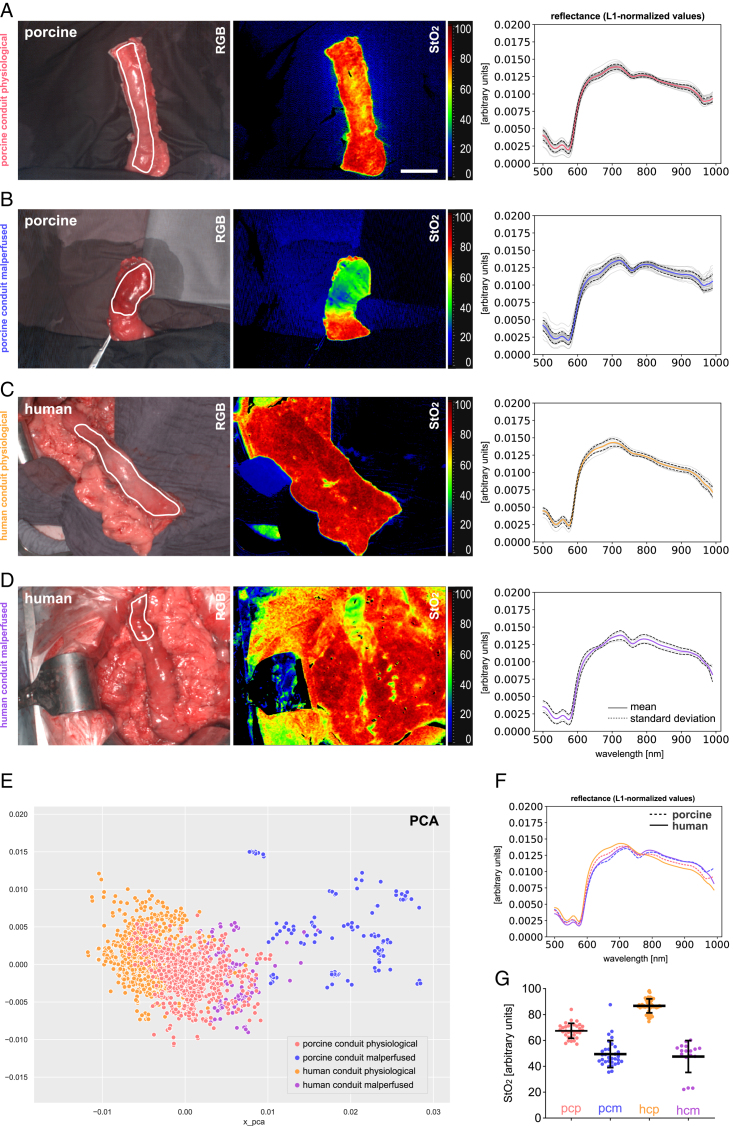
Comparison of human and porcine gastric spectra. HSI color index pictures and respective spectra for porcine and human gastric conduit. A, porcine gastric conduit physiological (pcp) (*n*=32, I=32). B, porcine gastric conduit malperfused (pcm) (*n*=32, I=32). C, human gastric conduit physiological (hcp) (*n*=57, I=10). D, human gastric conduit malperfused (hcm) (*n*=14, I=4). E, PCA visualization with an explained variability of 64.4% on the *x*-axis and 20.3% on the *y*-axis. F, comparison of L-1 normalized reflectance spectra. G, quantification of StO_2_ index values. Scale bar equals 5 cm.

Data from this porcine study was compared to human data from the SPACE trial (ZITAT) to foster the process of transferability (Fig. 7, Supplement Table 6, Supplemental Digital Content 10, http://links.lww.com/JS9/D43 and Supplement Figure 4, Supplemental Digital Content 11, http://links.lww.com/JS9/D44). In the inter-species comparison of L1-normalized spectra in Figure 7F, it could be seen that although slight differences exist, the overall curve shape of measured reflectance was highly comparable between the two species for the respective physiological and malperfused tissue states. The PCA with a cumulative explained variance of 85% indicates partial overlap between the clusters, especially for the two physiological groups. Both species showed a drop to comparable levels of oxygenation of 49.4% (±10.4%) and 47.6% (±12.3%) (Fig. 7G).

### Examples of clinical application

**Figure 8:**
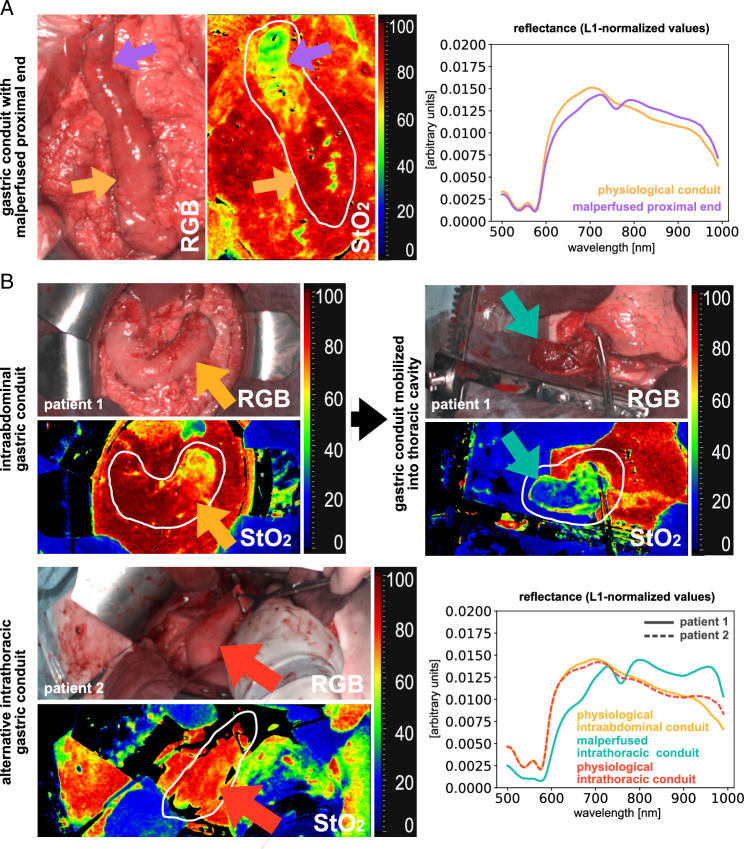
Examples of clinical application. Exemplary human data of single patients with useful clinical application. a, malperfused gastric conduit stump in the abdomen. b, gastric conduit before (physiological) and after (clearly malperfused) mobilization into the thoracic cavity compared to an intrathoracic physiological gastric conduit in another patient. Scale bar equals 5 cm.

During intraoperative HSI recordings in patients undergoing esophagectomy, further highly interesting situations could be captured that elucidate the potential of HSI and indicate possible future research questions (Fig. 8). Figure 8A depicts the intraoperative situation of gastric conduit malperfusion at the proximal end of the gastric stump. Figure 8B shows recordings of a gastric conduit before and after mobilization into the thoracic cavity. This mobilization led to impaired arterial perfusion through compression of the gastroepiploic artery and a subsequent clear demarcation of the conduit with a significant drop in oxygenation. In contrast, the gastric conduit of another patient is depicted with physiological levels of oxygenation after intrathoracic mobilization.

## Discussion

The imperative pursuit of identifying optimal therapeutic approaches for managing intraoperative hemorrhage during radical esophagectomy constitutes a paramount endeavor within the realm of surgical oncology. Hemorrhagic complications, if not swiftly and effectively addressed, can exert a profound influence on patient outcomes, exacerbating already high morbidity and mortality rates^1–5^. Thus, the cultivation and refinement of efficacious interventions aimed at mitigating intraoperative hemorrhage and evaluation organ perfusion represent a sine qua non for advancing patient safety and elevating surgical outcomes in this intricate surgical domain. Blood flow during surgical procedures is one if not the central aspect in surgery. Consequently, surgeons have been engaged in this topic for decades and have a variety of technical options to assess blood flow during surgery. The most established and currently practiced evaluation is probably still the visual and haptic feedback to the experienced surgeon. More direct objective and technological methods would include ultrasound Doppler, ICG-fluorescence, digital subtraction angiography, and Laser-speckle contrast imaging – or, as in this case, HSI – to name only a few.

Currently, resuscitation therapy subsequent to intraoperative hemorrhage is guided using surrogate parameters such as MAP or systemic lactate values. However, the variable that is actually of interest is the capillary perfusion and oxygenation on a microcirculatory level, as sufficient tissue oxygenation is vital and the most decisive factor for sustained physiological tissue function and integrity. HSI is exceptionally well-suited to evaluate oxygenation as it literally lets you watch microcirculation, which is the actual direct primary measure of interest for the surgeon.

In the present study, hemorrhagic shock caused dynamic changes in gastric conduit tissue oxygenation reliably detected by HSI, with StO_2_ values dropping from 68.5 to 62.5% across all four groups (Fig. 4). Similarly, Cohn *et al*.^27^ described a drop in oxygen saturation from 87 to 62% in a porcine hemorrhage model using a prototype oximeter fiberoptic probe. While their observed decrease in saturation was higher with 25%, the oximeter used by them was specialized for this single purpose providing digit readings instead of the multipurpose multidimensional HSI camera used in the present study.

Circulatory resuscitation was effective for volume-only and volume-catecholamine-combined regimes as indicated by HSI (Fig. 4). StO_2_ values of the gastric conduit showed significantly different results between the four experimental groups with 63.3% (±7.6%) after permissive hypotension (I), 45.9% (±6.4%) after catecholamine therapy (II), 70.5% (±6.1%) after crystalloid volume supplementation (III) and 69.0% (±3.7%) after combined therapy (IV). Comparable animal studies investigating hemorrhagic shock and resuscitation using crystalloid infusion (lactated Ringer’s solution) could show a similar increase of gastric oxygenation from 40% (±12%) to 59% (±4%) in the resuscitation group and a continued decrease over 51% (±17%) to 39% (±15%) StO_2_ in the control group after 2 h^27^. Clinical studies could already show a median mucosal oxygen saturation reduction of 38% after devascularization of the stomach as an even more extreme form of gastric malperfusion^28^ and that decreased gastric conduit oxygenation was highly associated with anastomotic complications^29^. The effect of catecholamine therapy on gastric tissue oxygen saturation after hemorrhage has not been part of experimental studies to our knowledge so far. The optimal therapeutic strategy to maintain blood pressure during esophagectomy with hemorrhage with regards to tissue perfusion of the gastric conduit as suggested by the current study would thus be crystalloid volume supplementation alone or in combination with catecholamines regarding short-term tissue oxygenation. This is underlined by the systemic lactate measurements in the current study. HSI measured StO_2_ values in the current study correlated strongly with systemic lactate values (r=−0.67; CI −0.77 to −0.54), which is an established prognostic factor. Similarly, Barberio *et al*.^15^ described a negative correlation factor of −0.54 for lactate and gastric StO_2_ values. However, they only focused on physiological gastric conduits und local capillary lactate, instead of systemic lactate measurements. In the present study, the measured StO_2_ values and the associated validity provided by lactate indicate that the purely catecholamine-based therapy regimen was inferior perioperatively, while crystalloid volume supplementation alone or in combination with catecholamines was the superior therapy regimen for maintaining blood pressure with regards to gastric conduit tissue oxygenation after hemorrhage during esophagectomy. This is important to consider since gastric conduit tissue perfusion and oxygenation are critical for anastomotic healing, which is, in turn, the single most decisive factor for perioperative outcome after esophagectomy. It has to be mentioned that the drastic differences in lactate values measured between the groups might also include dilution effects from the different amounts of i.v. fluids applied. However, the general statement that lactate levels are significantly greater in the control and catecholamine group holds firm.

Theoretically, the good correlation between StO_2_ and systemic lactate levels could be used as a prognostic predictor. However, this was not investigated in this trial and would need to be investigated in survival experiments with follow-ups.

There is still controversy regarding the particular effects of different catecholamine derivatives in different concentrations and different circulatory situations (normotension, septic shock, hemorrhagic shock, etc.) on different anatomical regions (splanchnic, peripheral, etc.)^30^. The benefits of elevation in systemic microcirculatory perfusion pressure are confronted with the disadvantages of capillary vasoconstriction and redistribution of blood flow away from the splanchnic region. What is certain is the potential of norepinephrine to also cause harm in higher dosage, for example, aggravating intestinal ischemia or reducing superficial tissue oxygenation^31,32^. And especially in the situation of hypovolemia after hemorrhage when physiological resources are sparse, catecholamines induce strong capillary vasoconstriction in visceral organs of the splanchnic region, including the stomach, depriving the organ of capillary flow and, therefore also of oxygen. This is known as the adrenergic centralization effect of catecholamines and was also shown in a clinical trial by Fischer *et al*.^33^ for patients experiencing hemorrhage. The use of catecholamines after the initial damage control surgery more than quadrupled colonic anastomotic leakage rates to 50%, yet no causality could be derived from this. A large randomized clinical trial by Poterman *et al*. investigated the effects of norepinephrine on peripheral oxygen saturation in patients undergoing elective general surgery under general anesthesia. They reported a decrease of 3% in peripheral oxygen saturation of the skin when norepinephrine was applied to counteract reduced MAP under general anesthesia^34^. Another animal study with a highly comparable experimental setup as ours evaluated perfusion of the gastric conduit after hemorrhage. Using solely systemic lactate/pyruvate ratio they could also confirm hypoperfusion of the conduit after the administration of noradrenaline with values changing from 511 to 792 L/p over just 1 h^35^. Therefore, it is comprehensible that norepinephrine appeared inferior to volume supplementation in a volume-depleted disease model possibly even aggravating the detrimental effects of volume depletion through excessive splanchnic vasoconstriction reducing oxygenation even further to only 45.9% and therefore even 17.4% less than the control group in the present study. The combination of catecholamine and volume resuscitation was not statistically different from volume resuscitation alone, but also yielded no advantages. Instead, the combined group provided tendencies of a slight decrease in StO_2_ values and simultaneously an increase in lactate readings in direct comparison (Fig. 6A). No comparable studies have been published addressing this direct comparison for hemorrhagic shock. Specific HSI patterns in the catecholamine group were indicative of the detrimental catecholamine-induced tissue hypoperfusion with a decrease in reflectance around 660 nm as seen in Figure 4E ^36,37^. Again, no reference measurements exist in the current literature.

Human data of 14 patients was comparable to porcine findings regarding oxygenation values and changes of reflectance spectra. Malperfusion of the distal gastric conduit in four patients could be clearly confirmed in the StO_2_ images (Fig. 7D). The conduits could be surgically shortened during the creation of the anastomosis, and no anastomotic insufficiencies occurred postoperatively. No comparable HSI data of human malperfused gastric conduit currently exists. While HSI seemed to be feasible for the detection of primary gastric conduit malperfusion in patients, we also depict its additional potential in the identification of secondary malperfusion, for example, due to kinking or torsion of the conduit after intrathoracic mobilization as an inspirational source for further trials (Fig. 8). The potential of HSI in solid cancer detection has already been shown for multiple entities^38–43^.

Limitations of this study include the lack of other interventional options and treatment concepts during hemorrhage, the limitation of volume application in patients, and the naturally given limitations of an animal study. Elaborating on the first limitation, this study has the aspiration of investigating the isolated and combined effects of crystalloid fluid and catecholamine resuscitation addressing hemorrhage during esophagectomy. Other concepts might include the application of hydroxyethyl starch, blood transfusions, or albumin application. However, hydroxyethyl starch has been subject to great controversies over recent years^44,45^, and blood transfusions have been linked to poorer long-term survival in patients undergoing esophagectomy, presumably due to its potential immunomodulatory effects^46^. The second limitation refers to the natural limitation of volume application in patients. While unrestricted volume transfusion might have beneficial effects on HSI tissue oxygenation in the perioperative short-term, it can impair anastomotic integrity through swelling of the anastomosis over the postoperative course due to inflammatory responses, osmotic barrier damage and secondary volume shifts^47^. This is a well-known mechanism that can cause anastomotic swelling with up to 25 mmHg lower bursting pressures of anastomoses and therefore has to be considered carefully. The multicenter randomized assessor-blinded RELIEF trial including 2983 patients undergoing major abdominal surgery could show significantly increased anastomotic leakage rate of 21% for liberal volume therapy of above 6000 ml on POD 0 and 22% for POD 4 versus 9 and 7% in case of restrictive volume therapy. Moreover, multivariate analysis confirmed fluid balance as independent predictor of anastomotic insufficiency^46,48^. Additionally, these numbers seem not to stand in isolation. Instead, also for elective colorectal resection a clinical trial could show tissue-healing complications of 16 versus 31% when applying restricted versus standard fluid regime^49^. Therefore, an extensive fluid application also cannot be the isolated solution of circulation resuscitation in hemorrhagic patients, but balance of volume replacement and catecholamine application will always be mandatory. This second limitation is associated with the third limitation that only a short perioperative window was evaluated as the animal models were only under observation for several hours and therefore could not experience inflammatory reactions and volume shifts as would be the case with patients over their first postoperative week. Regarding this final limitation, translating findings from animal experiments to human patients presents a complex puzzle, given the inherent disparities in anatomy, physiology, and genetics between species. Furthermore, human results are often more heterogenous as human application is difficult to standardize due to multiple factors and comorbidities relevant to microcirculation such as atherosclerosis, acute coronary syndrome, and type 2 diabetes mellitus. Although we do possess human data on record, which offers some insight into transferability, the challenges stemming from limitations in animal models, ethical constraints, and the inability to fully replicate the heterogeneity of human diseases still loom large. To effectively bridge this translational chasm, a meticulous, interdisciplinary approach is imperative. This approach involves harmonizing animal-derived findings with research methods centered on humans, such as clinical trials, to ensure the development of the most well-informed and practical therapeutic strategies. In the specific case of experimental animal models for esophagectomy, porcine gastric vascular anatomy is known to be highly comparable to human anatomy and an established model for experimental investigations^16,18^. For the purpose of this project’s main research question, the specific interest was the perfusion of the gastric conduit as one element of the anastomotic site in Ivor–Lewis esophagectomy, but also as a region representative of other visceral organs. Consequently, only the abdominal procedure of Ivor–Lewis esophagectomy was performed as a conventional gastric conduit formation with dissection of the inflow of the left gastroepiploic and left gastric arteries and resection of the lesser curvature^50^.

Ultimately, the choice for the most suitable intervention strategy for bleeding management during esophagectomy strongly depends on the specific case and has to be seen not only from the perspective of oxygenation, but on the counterside also with regards to intestinal edema. An anastomosis requires several conditions in order to heal including metabolism and oxygenation, but also mechanical aspects such as being free of tension and allowing endoluminal content to pass and not obstruct. This manuscript explicitly only focused on investigating isolated treatment effects on the oxygenation aspect. The volumes applied in this work are probably not feasible in real surgery as the anastomosis will be swollen and healing compromised. Much rather this work should simply raise awareness in the surgeon that catecholamines can actually have negative effects and avoiding crystalloid volume by any costs out of fear for edema by using excessive catecholamines to keep the MAP in the target area is not viable. We would therefore recommend a balanced approach with as much crystalloid volume as feasible monitored by regular consecutive therapy reevaluation, for example, using endoscopy to quantify mucosal edema. However, the optimal strategy to address hemorrhage is still to avoid it in the first place. Preoperative preparation and perioperative management in an experienced team that knows how to react in case of sudden bleeding – such as, for example, increasing pneumoperitoneum for venous bleeding or applying constant laparoscopic pressure while converting to open surgery for arterial bleeding – are crucial for this.

In summation, HSI microcirculatory monitoring could open up new opportunities for the guidance of haemodynamic management^18^. Laser Doppler flowmetry, near-infrared spectroscopy, laser speckle contrast imaging, fluorescence imaging, sidestream darkfield microscopy, and optical coherence tomography are alternative modalities that HSI will have to compete against in clinical studies^51^ as the pursuit of identifying optimal strategies for the management of intraoperative hemorrhage during radical esophagectomy remains an imperative quest for enhancing patient care.

However, it is incumbent upon the scientific community to remain circumspect regarding the inherent limitations posed by sole reliance on animal data. The future trajectory of research endeavors should aspire to insights gleaned from preclinical investigations with clinical evidence and require confirmation in human trials.

## Ethical approval

The research related to animal use complies with all the relevant national regulations, institutional policies and has been approved by the authors’ institutional review board or equivalent committee in Karlsruhe (G-261/19).

## Source of funding

There was no special funding for this project. Funding from the Willy Robert Pitzer Foundation (grant number: not applicable), the Heidelberg Foundation of Surgery (grant number: not applicable) and the European Research Council (ERC) under the European Union’s Horizon 2020 research and innovation program (NEURAL SPICING, Grant agreement No. 101002198) contributed to this work.

## Author contribution

A.S.-F.: conceptualization, methodology, software, validation, formal analysis, investigation, resources, data curation, writing – original draft, writing – review and editing, visualization, supervision, project administration, and funding acquisition; B.Ö.: software, investigation, and data curation; M.R. and L.A.: software, validation, and formal analysis; S.S.: software, validation, formal analysis, and investigation; J.S.: software, validation, formal analysis, and investigation; K.-F.K.: writing – review and editing, visualization, supervision, project administration, and funding acquisition; C.M.H.: investigation and data curation; J.O.: software and validation; S.K., D.G., and M.D.: investigation and data curation; K.S. and G.A.S.: investigation and writing – review and editing; F.S., A.D., H.N., T.H., and M.W.: writing – review and editing; B.P.M.-S.: conceptualization, resources, writing – review and editing, and supervision; L.M.-H.: conceptualization, methodology, software, resources, writing – review and editing, supervision, and project administration; F.N.: conceptualization, resources, writing – review and editing, supervision, project administration, and funding acquisition.

## Conflicts of interest disclosure

Felix Nickel reports support for courses and travel from Johnson and Johnson, Medtronic, Intuitive Surgical, Cambridge Medical Robotics and KARL STORZ as well as consultancy fees from KARL STORZ. The remaining authors report no conflicts of interest.

## Research registration unique identifying number (UIN)

Since this is an exploratory animal study, it has not been registered in a publicly accessible database.

## Guarantor

Felix Nickel.

## Data availability statement

The datasets used during this study are available from the corresponding author upon reasonable request.

## Supplementary Material

SUPPLEMENTARY MATERIAL
