# Supplementary figures and images for: Crystalloid volume versus catecholamines for management of hemorrhagic shock during esophagectomy: assessment of microcirculatory tissue oxygenation of the gastric conduit in a porcine model using hyperspectral imaging – an experimental study
Source: Int J Surg. 2024 Jul 8;110(10):6558–72. doi: 10.1097/JS9.0000000000001849 (PMC11486957; doi:10.1097/JS9.0000000000001849)

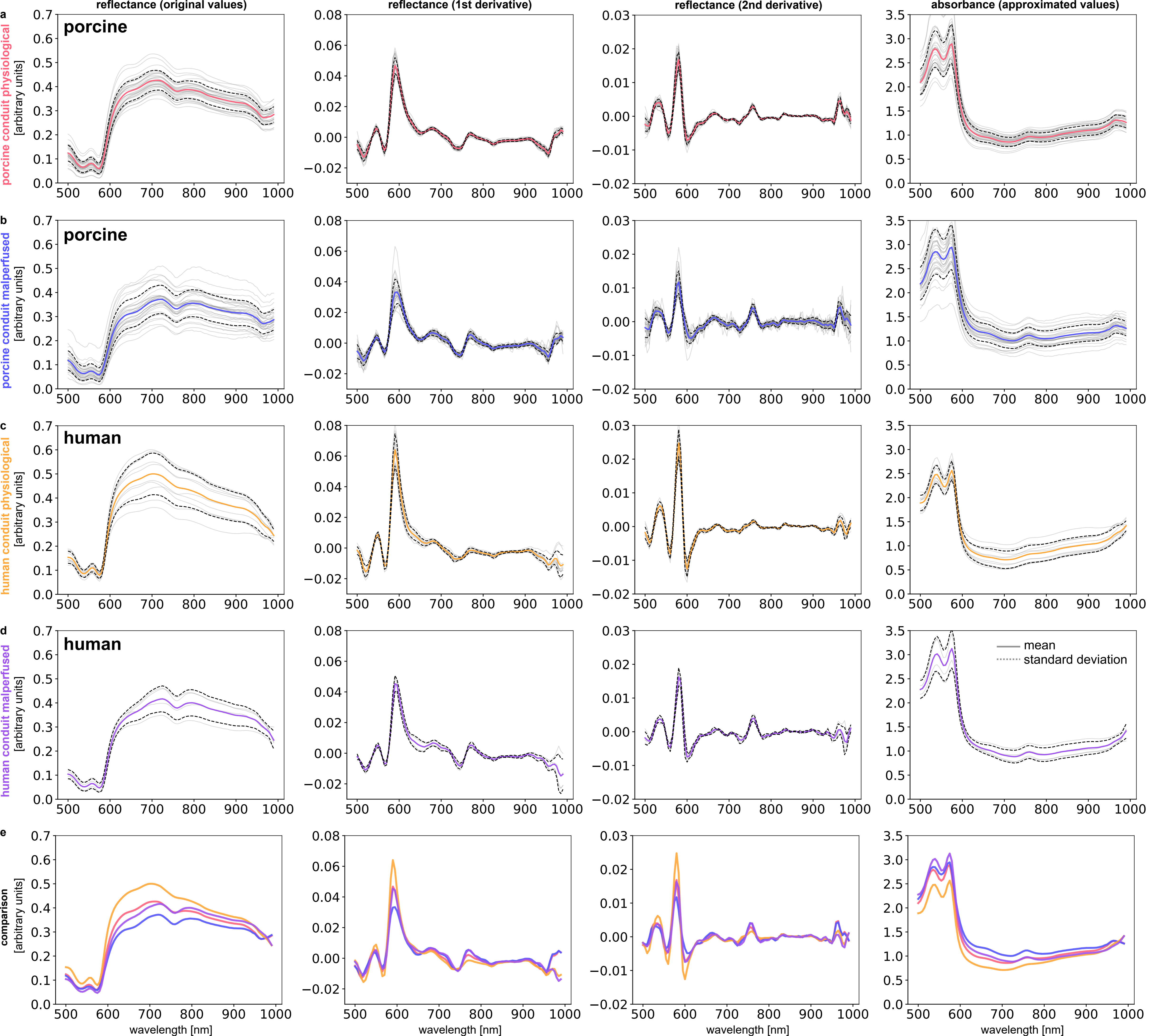

Supplement: SUPPLEMENTARY MATERIAL [file js9-110-6558-s003.pdf]

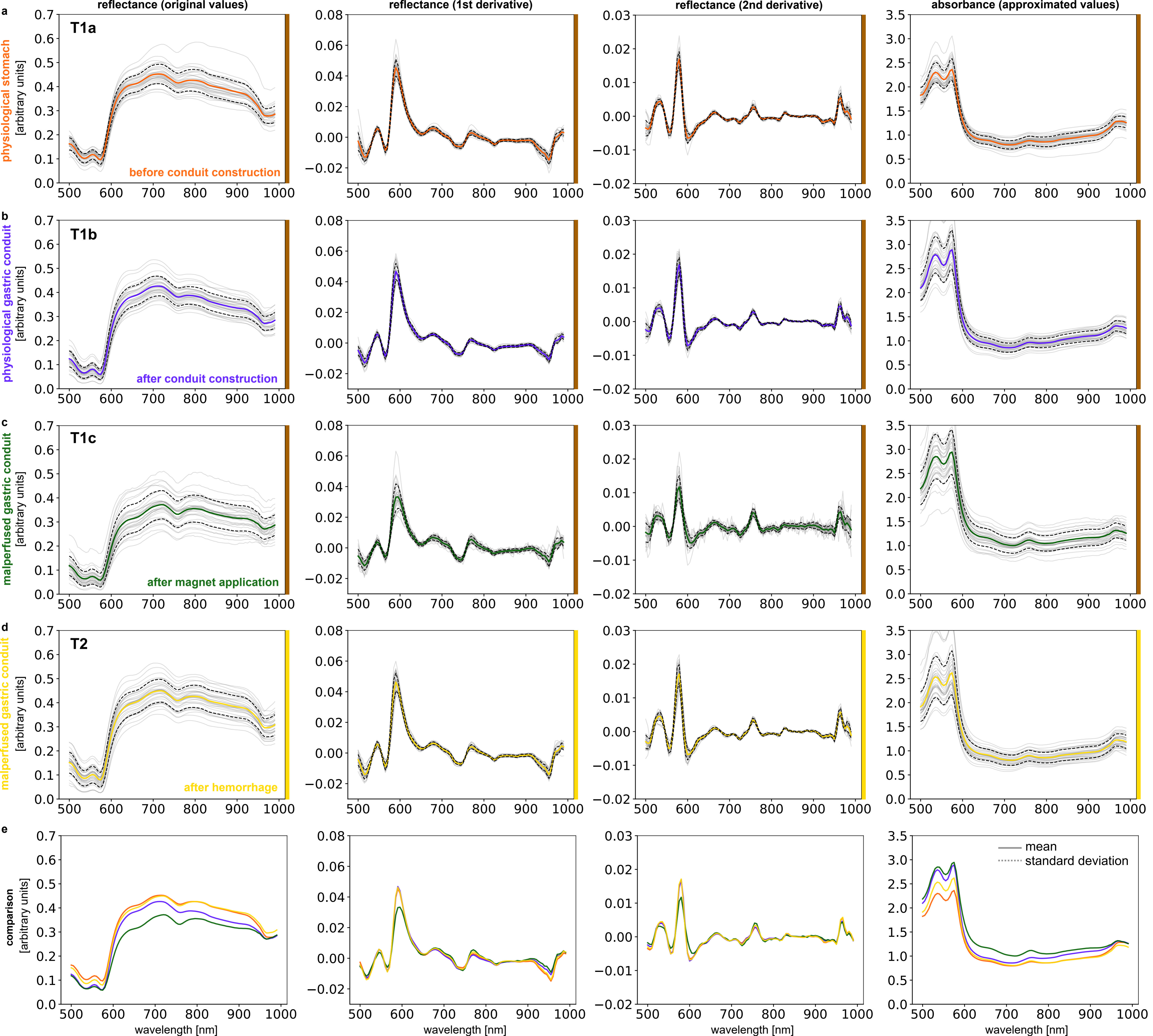

Supplement: SUPPLEMENTARY MATERIAL [file js9-110-6558-s005.pdf]

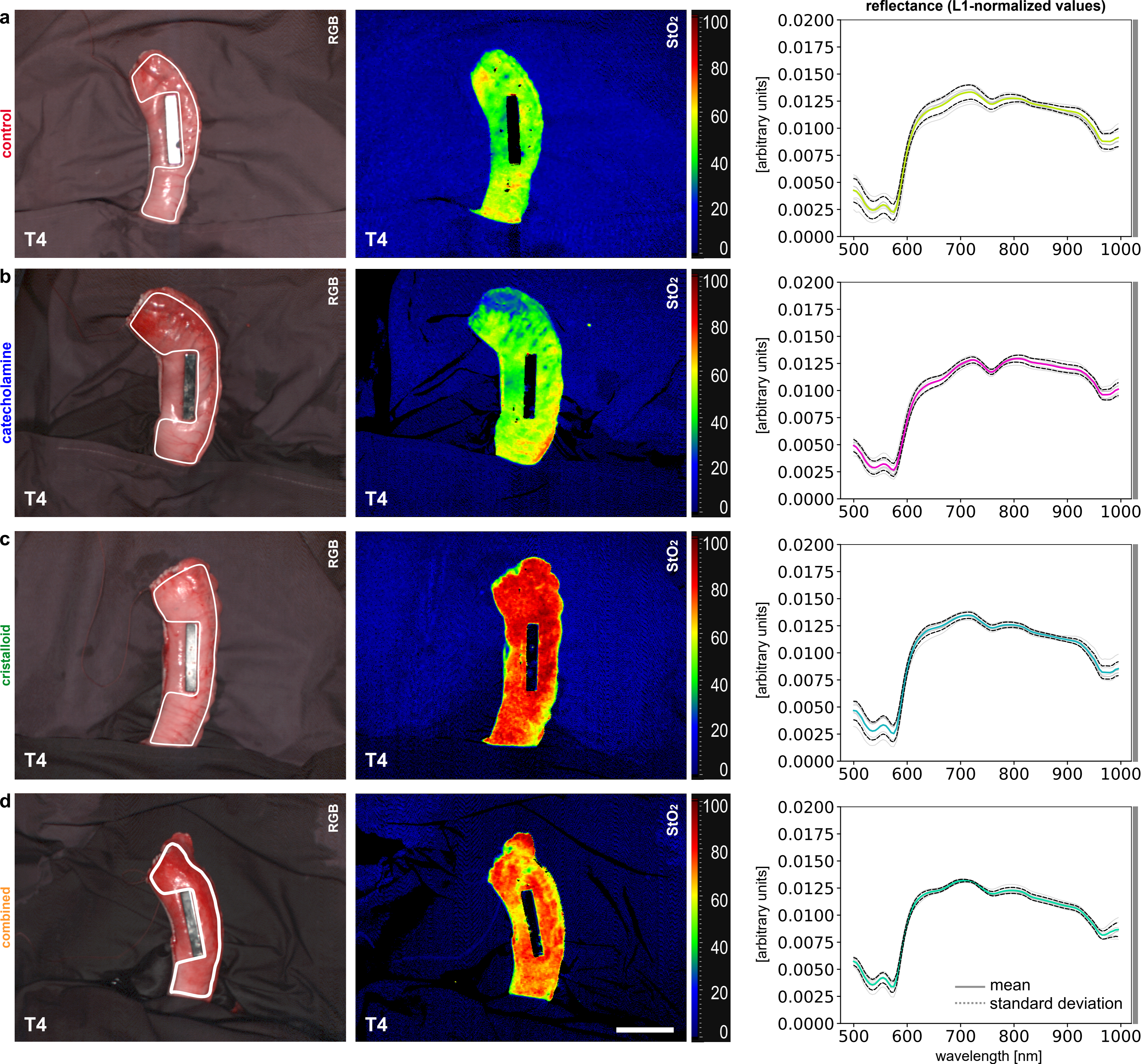

Supplement: SUPPLEMENTARY MATERIAL [file js9-110-6558-s008.pdf]

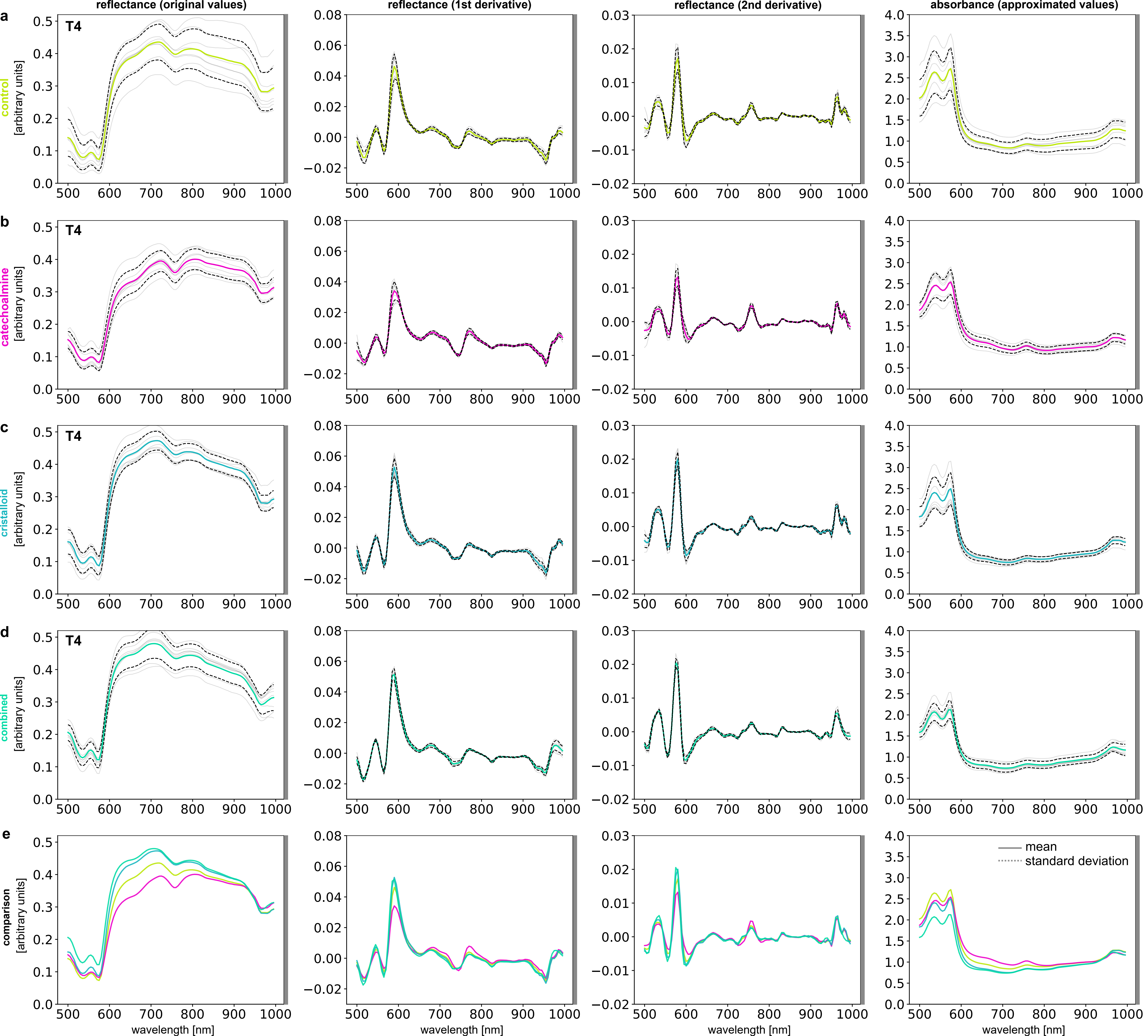

Supplement: SUPPLEMENTARY MATERIAL [file js9-110-6558-s009.pdf]
